# Independent and Joint Associations of Tea Consumption and Smoking with Parkinson’s Disease Risk in Chinese Adults

**DOI:** 10.3233/JPD-223148

**Published:** 2022-07-08

**Authors:** Jia Nie, Chunyu Liu, Canqing Yu, Yu Guo, Pei Pei, Ling Yang, Yiping Chen, Huaidong Du, Kaifei Zhu, Danile Schmidt, Daniel Avery, Junshi Chen, Zhengming Chen, Jun Lv, Liming Li, for the China Kadoorie Biobank (CKB) Collaborative Group

**Affiliations:** a Department of Epidemiology & Biostatistics, School of Public Health, Peking University, Beijing, China; b Peking University Center for Public Health and Epidemic Preparedness & Response, Beijing, China; c Fuwai hospital, Chinese Academy of Medical Sciences, Beijing, China; d Chinese Academy of Medical Sciences, Beijing, China; e Medical Research Council Population Health Research Unit at the University of Oxford, Oxford, United Kingdom; f Clinical Trial ServiceUnit & Epidemiological Studies Unit (CTSU), Nuffield Department of Population Health, University of Oxford, United Kingdom; g NCDs Prevention and Control Department, Wuzhong Center for Disease Control and Prevention, China; h China National Center for Food Safety Risk Assessment, Beijing, China; i Key Laboratory of Molecular Cardiovascular Sciences (Peking University), Ministry of Education, Beijing, China

**Keywords:** Chinese, Parkinson’s disease, prospective study, smoking, tea consumption

## Abstract

**Background:**

Existing limited evidence suggests that smoking and tea consumption may be associated with a lower risk of Parkinson’s disease (PD). However, less is known about the independent and joint roles of these two habits, which are often clustered among Chinese, on PD risk.

**Objective:**

To prospectively examine the independent and joint association of tea consumption and smoking with the risk of PD.

**Methods:**

The China Kadoorie Biobank (CKB) study recruited 512,725 participants aged 30 to 79 years from ten areas across China since 2004. Information on smoking and tea consumption was collected at baseline, and PD cases were ascertained by linkage to the national health insurance system and death registry. Cox proportional hazards models were used to estimate the multivariable-adjusted hazard ratios (HRs) and corresponding 95%confidence intervals (CIs).

**Results:**

During a median of 10.8 years of follow-up, 922 PD cases were recorded. Compared with participants who never consumed tea, the HR (95%CI) for daily consumers was 0.68 (0.55, 0.84). Compared with participants who never or occasionally smoked, the HR (95%CI) for current smokers was 0.66 (0.53, 0.82). Those who had a clustering habit of smoking and tea consumption had a 38%(HR = 0.62; 95%CI: 0.49, 0.79) lower PD risk than those who consumed none. However, there were no statistically significant multiplicative or additive interaction for tea consumption and smoking on PD risk.

**Conclusion:**

We found that smoking and daily tea consumption were independently inversely associated with the risk of PD.

## INTRODUCTION

Parkinson’s disease (PD) is a common neurodegenerative disorder in middle-aged and older crowds; its etiology is not well understood [1]. The number of individuals with PD has more than doubled over 26 years, from 2.5 million patients in 1990 to 6.1 million in 2016 [2], imposing a heavy burden on society and families. Previous meta-analyses, including principally case-control studies, reported that tobacco smoking is a protective factor of PD [3, 4]. However, one of the important limitations of such a retrospective case-control study design is the big chance of reverse causality bias. The prediagnostic phase of PD is likely to be very long, during which time patients with PD quit smoking more easily than controls [5]. The few available prospective studies were primarily conducted in Western populations and with the small number of cases [6–11].

Tea is a popular beverage consumed all over the world [12]. Several experimental studies had demonstrated that the natural bioactive components in tea, such as polyphenols, theanine, and caffeine may bring neuroprotective effects on neurodegenerative diseases such as PD [13, 14]. A meta-analysis including four case-control and four prospective studies reported a linear relationship between tea consumption and decreased PD risk, and so as the pooled results from prospective studies only [15]. However, there is scant prospective evidence for the Chinese population, for which there is a long tradition of tea consumption. Also, the clustering of tea consumption and smoking is common in Chinese males. Any study on the association of PD with smoking or tea consumption in the Chinese population needs carefully control for confounding from each other.

In the China Kadoorie Biobank (CKB) study of 0.5 million adults, we aim to prospectively examine the independent and joint association of tea consumption and smoking with the risk of PD.

## METHODS

### Study population

The detailed information of the CKB and baseline characteristics of the participants have been described previously [16, 17]. Briefly, between 2004 and 2008, the CKB study recruited 512,725 participants aged 30 to 79 years from five urban and five rural areas across China. At recruitment, trained investigators conducted face-to-face interviews using an electronic questionnaire and standard physical measurements after participants provided written informed consent. This study was approved by the ethical committees of the Chinese Centre for Disease Control and Prevention and the University of Oxford.

### Assessment of tea consumption and smoking

Our baseline questionnaire collected tea consumption information, including frequency, amount of tea leaves consumed, types of tea consumed (green/jasmine tea, oolong tea, black tea, other tea), and the ages at which participants started drinking tea regularly (the detailed grouping information see Supplementary Material 2). For current analyses, we divided participants into three frequency groups: those who never drank tea, those who drank tea but less than daily or had a period of at least one year during which they drank tea at least once a week (hereinafter referred to as “less than daily”), and daily tea consumers. For daily consumers, we further categorized participants according to the amount of tea leaves consumed (≤ 3.0 and > 3.0 grams per day), duration of consumption (< 25 and ≥ 25 years), and types of tea consumed (green tea and others).

Information on smoking was collected at baseline, including frequency, type, and amount of tobacco smoked per day, ages since starting to smoke for ever smokers, and years since quitting for former smokers (the detailed grouping information, see Supplementary Material 2). Based on these questions, we divided participants into three frequency groups: those who never or occasional smoked, former smokers, and current smokers. Among current smokers, the conversion from tobacco to an equivalent in cigarettes was well described previously [18]. We further categorized participants according to the amount of tobacco smoked (1–19 and ≥ 20 cigarettes or equivalent per day), duration of smoking (< 30 and ≥ 30 years) among current smokers, and years since stopped smoking among former smokers (< 6 and ≥ 6 years) according to the median years.

### Assessment of covariates

Covariate information for all participants was collected by baseline questionnaire, including sociodemographic characteristics (age, sex, education), lifestyle behaviors (physical activity, alcohol consumption, intakes of dairy products, red meat, fresh fruits, and vegetables), and medical history. Physical activity level was quantified by summing the metabolic equivalent tasks (METs) value for specific activities with hours spent on as weights. According to standard measurement procedures, the trained investigators took physical measurements, including height, weight, and blood pressure. A random venous blood sample was collected for storage and the plasma glucose concentration was measured on-site (SureStep Plus system, LifeScan). Prevalent diabetes was defined as blood glucose ≥ 7.0  mmol/L with time since last food/beverage ≥ 8 h or ≥ 11.0 mmol/L with time since last food/beverage < 8 h, or self-reported diagnosis of diabetes. Prevalent hypertension was defined as measured systolic blood pressure ≥ 140 mm Hg, measured diastolic blood pressure ≥ 90 mm Hg, self-reported diagnosis of hypertension, or use of anti-hypertensive agents at baseline.

### Ascertainment of study outcome

The outcome of participants was ascertained through linkage to the local Disease Surveillance Points (DSP) system, disease and death registries, and national health insurance (HI) system since the enrollment, and was coded according to ICD-10 by trained medical staff who did not know the baseline information. PD events were coded as G20. Participants who had a PD diagnosis appeared in death certificates or hospitalization records were regarded as having the outcome.

### Statistical analyses

For the present analysis, we excluded participants with missing body mass index (BMI) (*n* = 2) at baseline, and a total of 512,723 participants were included. We counted person-years at risk for each participant from baseline (2004–2008) to the date of outcome documented, death, loss to follow-up, or 31 December 2017, whichever occurred first. Either multiple linear regression or logistic regression was used to assess the distribution of baseline characteristics of participants according to jointly grouped tea consumption and smoking status.

Cox proportional hazard models were used to examine the independent and joint associations of tea consumption and smoking with risk of PD, estimating hazard ratios (HRs) and their 95%confidence intervals (CIs). The models used chronological age as the underlying time scale and were stratified jointly by sex, ten regions, and age at baseline in 5-year intervals. For the analysis between tea consumption and the risk of PD, we used participants who never consumed tea as the reference group. The multivariable models were adjusted for age (years), education (no formal school, primary school, middle school, high school, college, or university or higher), alcohol intake (non-drinker, former drinker, weekly drinker, daily drinker: < 15, 15–29, 30–59, or ≥60 g/day of pure alcohol), intake of dairy products (day/week; calculated by assigning participants to the midpoint of their consumption category), level of physical activity (MET-hour/day), BMI (kg/m^2^), prevalent hypertension and diabetes (yes or no), and smoking status (non-smoker, former smoker, current smokers: 1–14, 15–24, or ≥25 cigarettes or equivalent/day). For the analysis of the association between smoking and the risk of PD, we used participants who never or occasionally smoked as the reference group. The models were adjusted for the same covariates but replacing smoking status with tea consumption (never, less than daily, daily tea drinker: ≤2.0, 2.1–4.0, > 4.0 leaf grams per day). Analyses were repeated in men and women separately. *p*-values for trends across categories were calculated where appropriate.

To minimize possible reverse causation or confounding bias, we further conducted sensitivity analysis by: 1) excluding cases documented during the first five years of follow-up, 2) excluding participants with prevalent cancer, stroke, or coronary heart diseases at baseline, 3) excluding participants with a medical history of head injury at baseline, 4) further adjusting for intake of meat, fresh fruits, and vegetables, or 5) further adjusting for the duration of storing pesticide at home.

Further, we conducted subgroup analyses according to baseline characteristics, including age, region, education, alcohol consumption, dairy products consumption, physical activity, BMI, prevalent hypertension, and diabetes. Likelihood ratio tests were used to compare models with and without a cross-product term.

To more strictly control for confounding caused by tea consumption or smoking, we restricted the analyses of association between tea consumption and risk of PD to participants who never or occasionally smoked, or to those who never consumed tea in life when concerned about smoking. In the joint association analyses, we divided participants into four groups according to the joint status of ever smoking (yes or no) and ever tea-drinking (yes or no). Multiplicative interaction between smoking and tea drinking on PD risk was assessed by adding a multiplicative interaction term to the model and using the likelihood ratio test. Additive interaction was assessed by estimating the relative excess risk due to interaction (RERI) [19, 20]. A RERI of 0 indicates no interaction on the additive scale, and > 0 indicates a synergistic interaction.

We used Stata version 15.0 (StataCorp, TX, USA) for statistical analyses and R (version 4.0.2) for plots. *p* values were two-sided and statistical significance was defined as *p* < 0.05.

## RESULTS

Of all the 512,723 participants included at baseline, the mean age was 52.0±10.7 years, 41%were male, and 44.1%resided in urban areas. Among participants included, 74.4%of males and 3.2%of females reported current or former smoking, 40.5%of males and 15.9%of females reported daily tea drinking. For male participants who were current or former smokers, 84.1%drank tea, and 45.7%reported daily tea drinking. For female smokers, 61.2%drank tea and 16.0%reported daily tea drinking.

Among current or former smokers, those tea consumers reported a higher amount of tobacco smoked per day compared with those who never consumed tea in life (Table 1). Also, among those tea consumers, smokers reported consuming a higher amount of tea leaves and a longer duration of tea consumption compared with those who never or occasionally smoked. The proportion of daily alcohol drinkers was higher among current or former smokers than never smokers.

**Table 1:** Baseline characteristics of participants according to smoking and tea consumption (*n* = 512,723)

	Smoking (–) & tea-drinking (–)	Smoking (+) & tea-drinking (–)	Smoking (–) & tea-drinking (+)	Smoking (+) & tea-drinking (+)
No. of participants, n (%)	148,581 (29.0)	28,636 (5.6)	198,059 (38.6)	137,447 (26.8)
Female, %	90.2	13.3	80.1	4.4
Age, y	52.7	55.8	50.5	52.7
Urban, %	44.8	40.0	46.3	41.1
Middle school or higher, %	45.7	40.1	54.6	47.0
Tobacco smoking
Amount smoked per day, cigarette or equivalent
Male	–	17.0	–	18.6
Female	–	9.3	–	9.8
Duration of smoking, y
Male	–	30.5	30.3
Female	–	34.0	32.4
Tea consumption
Tea leaves add per day, g	–	–	3.5	4.4
Duration of consumption, y	–	–	23.0	24.7
Green tea consumers, %	–	–	86.1	84.6
Daily alcohol drinker, %
Male	8.4	18.8	12.2	24.5
Female	0.5	1.8	0.9	3.5
Consumption of dairy products ≥4 days/week, %	10.6	8.8	14.0	11.0
Physical activity, MET-h/d	21.1	21.9	21.0	21.1
BMI, kg/m^2^	23.6	23.0	24.0	23.5
Prevalent hypertension, %	35.6	32.1	36.2	34.2
Prevalent diabetes, %	5.8	5.4	6.0	6.1

BMI, body mass index; MET, metabolic equivalent of task. Smoking (–): never or occasional smoking; tea-drinking (–): never tea-drinking; smoking (+): current or former smoking; tea-drinking (+): current or former tea drinking. Values are means or percentages and were adjusted for age, sex, and region, where appropriate.

### Tea consumption and risk of PD

**Fig. 1:**
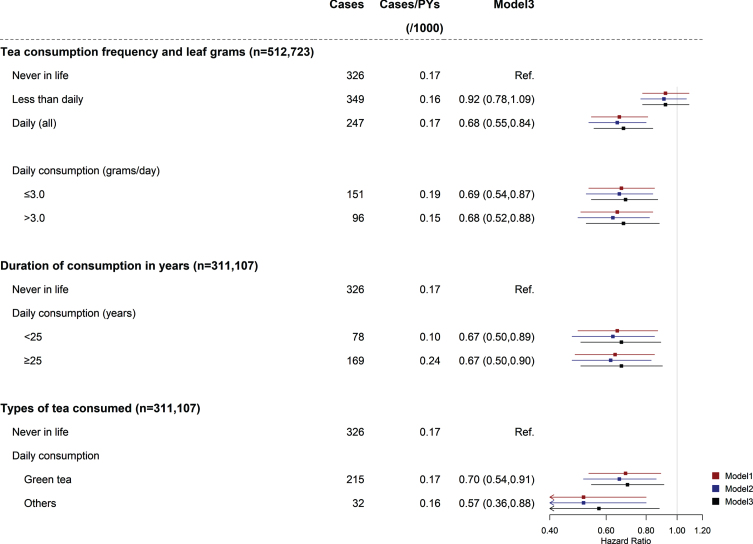
Association between tea consumption and risk of PD. Values were obtained from a Cox proportional hazards analysis. Multivariable analyses were adjusted for age and education for Model1. Model 2 was further adjusted for alcohol intake, intake of dairy products, level of physical activity, body mass index, prevalent diabetes, and prevalent hypertension. Model 3 was further adjusted for smoking. Square dots represent the HRs and horizontal lines represent the corresponding 95%CIs. Unadjusted incidence rates are reported per 1000 person-years of follow-up. PD, Parkinson’s disease; PYs, person years.

During 5,548,934 person-years (median, 10.8 years) of follow-up, there were 922 PD cases recorded. The mean age of documented PD cases was 68.5±9.2 when they were first documented with PD during the follow-up. The risk of PD was inversely associated with tea consumption (Fig. 1). Such association was slightly attenuated when we further adjusted for the status of smoking (model2 to model3). As compared with participants who never consumed tea in life, the fully adjusted HRs (95%CIs) for less than daily consumers and daily consumers were 0.92 (0.78, 1.09) and 0.68 (0.55, 0.84). However, the PD risk did not decrease with the increasing amount of tea leaves consumed or duration of consumption in years among daily tea consumers (model3, both *p*_trend_ > 0.05). Compared with those who never consumed tea in life, the HRs (95%CIs) for daily consumers who preferred green tea and non-green tea were 0.70 (0.54, 0.91) and 0.57 (0.36, 0.88), respectively.

Associations between tea consumption and the risk of PD were consistent between men and women (*p*_int_ > 0.05 for sex) (Supplementary Table 1). Sensitivity analyses showed no substantial changes in the results (Supplementary Table 2). There were no clinically meaningful differences across different groups of baseline characteristics (Supplementary Table 3).

### Smoking and risk of PD

**Fig. 2:**
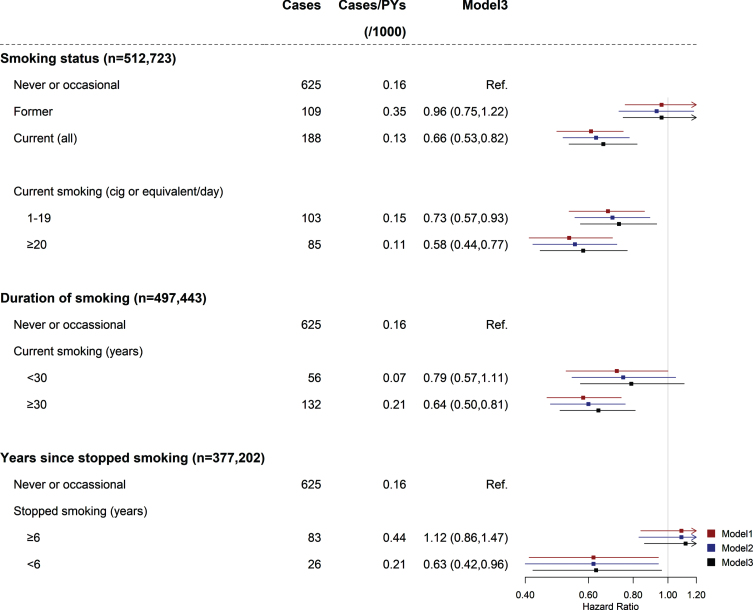
Association between smoking and risk of PD. Values were obtained from a Cox proportional hazards analysis. Multivariable analyses were adjusted for age and education for Model1. Model 2 was further adjusted for alcohol intake, intake of dairy products, level of physical activity, body mass index, prevalent diabetes, and prevalent hypertension. Model 3 was further adjusted for tea consumption. Square dots represent the HRs and horizontal lines represent the corresponding 95%CIs. Unadjusted incidence rates are reported per 1000 person-years of follow-up. PD, Parkinson’s disease; PYs, person years.

The inverse association between smoking and PD risk was also slightly attenuated when we further adjusted for tea consumption (model2 to model3) (Fig. 2). Compared with participants who never or occasionally smoked, the fully adjusted HRs (95%CIs) for former smokers and current smokers were 0.96 (0.75, 1.22) and 0.66 (0.53, 0.82). The inverse association was stronger when the amount of tobacco smoked increased as the HRs (95%CIs) for current smokers who smoked < 20 and ≥20 cigs or equivalent per day were 0.73 (0.57, 0.93) and 0.58 (0.44, 0.77) (model3, *p*_trend_ = 0.146), respectively. Compared with participants who never or occasionally smoked, the HRs (95%CIs) for those who smoked < 30 years and ≥30 years were 0.79 (0.57, 1.11) and 0.64 (0.50, 0.81) (model3, *p*_trend_ = 0.111). The HRs (95%CIs) were 1.12 (0.86, 1.47) and 0.63 (0.42, 0.96) for those who stopped smoking ≥6 years and < 6 years compared with those who never or occasionally smoked, respectively (model3, *p*_trend_ = 0.017).

Associations between smoking and the risk of PD were consistent between men and women (*p*_int_ > 0.05 for sex) (Supplementary Table 4). The results were not altered in sensitivity analyses (Supplementary Table 5). The associations were consistent across all subgroups stratified by potential baseline risk factors (p_int_ > 0.05) (Supplementary Table 6).

### Independent and joint associations of tea consumption and smoking with risk of PD

**Fig. 3:**
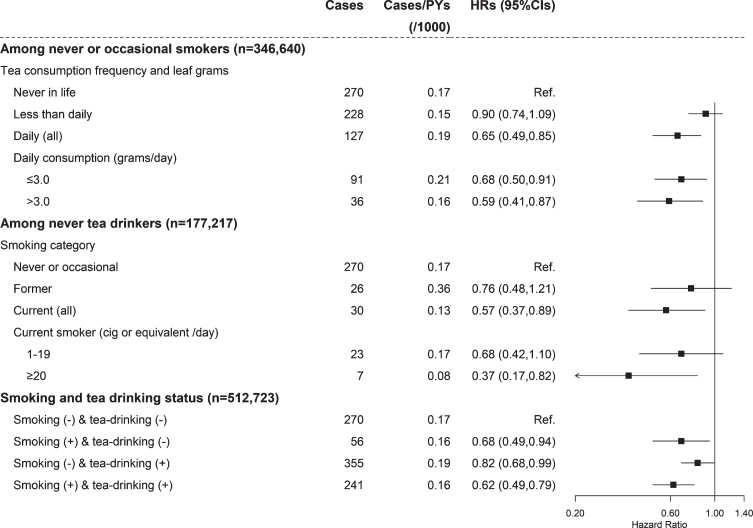
Independent and joint associations of tea consumption and smoking with PD risk. Values were obtained from a Cox proportional hazards analysis. Multivariable analyses were adjusted for the same covariates as model2 in Fig. 1. Smoking (–): never or occasional smoking; tea-drinking (–): never tea-drinking; smoking (+): current or former smoking; tea-drinking (+): current or former tea drinking. Square dots represent the HRs and horizontal lines represent the corresponding 95%CIs. Unadjusted incidence rates are reported per 1000 person-years of follow-up. CI, confidence interval; HR, hazard ratio; PD, Parkinson’s disease; PYs, person years.

When restricting analyses to participants who never or occasionally smoked, the adjusted HRs (95%CIs) for less than daily consumers and daily consumers who consumed ≤ 3.0 and  > 3.0 grams/day were 0.90 (0.74, 1.09), 0.68 (0.50, 0.91), and 0.59 (0.41, 0.87) (*p*_trend_ = 0.746 for daily consumers) (Fig. 3). When restricting analyses to participants who never drank tea, the adjusted HRs (95%CIs) for former smokers and current smokers who smoked < 20 and ≥20 cigs or equivalent per day were 0.76 (0.48, 1.21), 0.68 (0.42, 1.10), and 0.37 (0.17, 0.82) (*p*_trend_ = 0.069 for current smokers).

Compared with participants who neither smoked nor drank tea, the adjusted HRs (95%CIs) for those who were current or former smokers but did not drink tea, those who never or occasionally smoked but were ever tea drinkers, and those who both smoked and drank tea were 0.68 (0.49, 0.94), 0.82 (0.68, 0.99), and 0.62 (0.49, 0.79), respectively. The multiplicative interaction for tea consumption and smoking were not statistically significant (*p*_int_ = 0.561), either the addictive interaction [RERI: 0.12 (–0.39, 0.63)].

## DISCUSSION

This study provides prospective evidence on the independent and joint associations of tea consumption and smoking with the risk of PD in a large, well-established Chinese cohort. Daily tea consumption was independently inversely associated with PD risk. Also, the PD risk was reduced with the increased amount of tobacco smoked after control for tea consumption. Those who had clustering habits of smoking and tea consumption had a 38%lower risk of PD compared with those who consumed none. However, there were no statistically significant multiplicative or additive interactions for tea consumption and smoking on PD risk.

### Comparison with other studies and potential mechanism

Several prospective studies explored the relationship between smoking and the risk of PD. A European population-based cohort study of 220,494 participants recorded 715 PD cases after a median of 12.8 years follow-up [8], reporting the HRs (95%CIs) of PD risk for former smokers and current smokers were 0.79 (0.66, 0.94) and 0.49 (0.38, 0.63) compared with never smokers. For former smokers, the reverse association between smoking and PD remained until 18 years after quitting smoking. The associations of smoking intensity or duration with PD risk found in this study were also similar to our findings. Another study based on the British Doctor cohort of 29,737 males reported 283 deaths with PD as their underlying cause of death after an average follow-up of 35 years [6]. Compared with never smokers, they found that the HRs (95%CIs) of PD risk for former smokers and current smokers were 1.11 (0.87, 1.41) and 0.71 (0.60, 0.84). Among former smokers, the HRs (95%CIs) of PD risk for those who quit smoking 0–9 years and ≥10 years were 0.71 (0.54, 0.93) and 0.86 (0.70, 1.06). Both these two previous studies and current study found the reduced PD risk of current smoking, and also, with the increasing years since stopped smoking, the protective effect faded, although there were differences in the duration of years in which the effect of smoking persisted.

Only a few prospective cohort studies assessed the association between tea consumption and PD risk and showed inconsistent findings [21–24]. A prospective study of 29,335 Finnish participants aged 25 to 74 years with a mean follow-up of 12.9 years reported 200 incident PD cases. The results showed that compared with non-drinkers, the HR (95%CI) for participants daily drinking ≥3 cups of tea was 0.41 (0.20, 0.83) [21]. The other two, based on the Health Professionals’ Follow-Up Study, the Nurses’ Health Study and the CPS II-Nutrition Cohort, reported that tea consumption was not statistically associated with PD risk, more likely due to a small number of cases and insufficient power [22, 23]. The Singapore Chinese Health Study (SCHS) of 63,257 Chinese participants identified 157 incident PD cases at follow-up interviews on average 7 years after enrollment [24]. This study showed that black tea drinking was associated with a reduced risk of PD, but the association was not statistically significant in green tea consumers. Compared with nondrinkers, the related risks (RRs) for black tea consumers who drank < 5, 5–22, ≥23 cups/month were 0.87 (0.54, 1.40), 0.54 (0.29, 1.00) and 0.29 (0.13, 0.67) (*p*_trend_ < 0.05), respectively. In contrast, in the present CKB population, daily tea consumption was inversely associated with PD risk, but the risk did not decrease with the increasing amount of tea leaves consumed or duration of consumption among daily consumers. Also, the reduced risk of PD appeared in both daily green and non-green tea consumers.

The associations of smoking or tea consumption with reduced risk of PD are biologically plausible. Clinical and experimental evidence suggests that nicotine in smoke can protect dopamine neurons via regulating striatal activity and behaviors mediated through the dopaminergic system [25]. It has also been reported that tea polyphenols can significantly suppress dopamine-related toxicity via multiple pathways such as inhibition of dopamine oxidation, conjugation with dopamine quinones, and scavenge of reactive oxygen species [26].

The SCHS reported the combined effects of black tea consumption and smoking with PD risk. Compared with those who were non-tea-drinkers and non-smokers, the RRs (95%CIs) of PD for those non-tea-drinkers and smokers, those tea-drinkers and non-smokers, and those both tea-drinkers and smokers were 0.48 (0.31, 0.75), 0.57 (0.35, 0.93), and 0.33 (0.16, 0.68). In the present CKB population, with green tea the most popular drink, we had consistent findings. However, our further tests showed no statistically significant additive or multiplicative interaction between tea consumption and smoking on PD risk.

### Strengths and limitations of this study

This large-scale prospective cohort study of the Chinese population provides robust evidence on the independent and joint associations of smoking and tea consumption with PD risk. The strengths of the study include its population-based prospective design, a widespread population of different sociodemographic characteristics across China, a relatively large number of cases, and extended follow-up. The detailed baseline information collection enabled us to assess several metrics of tea consumption, including the frequency, amount, duration, and types of tea consumed, as well as the habit of smoking. Also, we carefully adjusted the potential confounders, such as other kinds of lifestyle habits and disease histories.

Inevitably, there are some limitations. Firstly, information on tea consumption and smoking was self-reported, which might raise the possibility of misclassification. Information on tea consumption and smoking was collected once at baseline, although these two habits are more likely to be habitual. Second, a proportion of PD cases might not have been detected during follow-up because most of the cases were ascertained from hospitalization records of the health insurance database. So the present findings may be carefully explained as the associations of PD cases with overt symptoms who need intensive medical care or were hospitalized for other conditions with tea consumption and smoking. Besides, given the complexity of PD diagnosis, it is difficult to review each PD case one by one in such a large-scale long-term follow-up study. Thus the diagnostic accuracy in the current study remained uncertain, and there might be diagnostic misclassification. Third, we did not collect PD history at baseline so we could not exclude few but possible PD cases in advance. Despite an attempt to exclude PD cases documented in the first five years of follow-up in the sensitivity analysis, as a fact of the long prediagnostic phase of PD, we still could not rule out the reverse causality bias completely.

### Conclusion

Overall, in this large-scale, long-term follow-up study of Chinese adults, we found that smoking and daily tea consumption were independently inversely associated with the risk of PD. We could not make a causal inference because of the limitation of the observational study. More large prospective studies in different populations, especially with longer follow-up time and enhanced capture of PD cases, are warranted to replicate the findings. The mechanisms underlying these inverse associations also demand further research. If the associations of smoking and tea consumption with PD were causal, then efforts to identify the bioactive substances may help ease the burden of PD.

## Supplementary Material

Supplementary Material

## ACCESS TO RESEARCH MATERIALS

Details of how to access China Kadoorie Biobank data and details of the data release schedule are available from https://www.ckbiobank.org/site/Data+Access.
