# Supplementary material for: Independent and Joint Associations of Tea Consumption and Smoking with Parkinson’s Disease Risk in Chinese Adults
Source: J Parkinsons Dis. 2022 Jul 8;12(5):1693–702. doi: 10.3233/JPD-223148 (PMC9398092; doi:10.3233/JPD-223148)
Supplement: Supplementary Material [file jpd-12-jpd223148-s001.pdf]

Supplementary Material

Independent and Joint Associations of Tea Consumption and Smoking with Parkinson's Disease Risk in Chinese Adults

Supplementary Material 1. Members of the China Kadoorie Biobank collaborative group: **International Steering Committee:** Junshi Chen, Zhengming Chen (PI), Robert Clarke, Rory Collins, Yu Guo, Liming Li (PI), Jun Lv, Richard Peto, Robin Walters. **International Co-ordinating Centre, Oxford:** Daniel Avery, Ruth Boxall, Derrick Bennett, Yumei Chang, Yiping Chen, Zhengming Chen, Robert Clarke, Huaidong Du, Simon Gilbert, Alex Hacker, Mike Hill, Michael Holmes, Andri Iona, Christiana Kartsonaki, Rene Kerosi, Ling Kong, Om Kurmi, Garry Lancaster, Sarah Lewington, Kuang Lin, John McDonnell, Iona Millwood, Qunhua Nie, Jayakrishnan Radhakrishnan, Paul Ryder, Sam Sansome, Dan Schmidt, Paul Sherliker, Rajani Sohoni, Becky Stevens, Iain Turnbull, Robin Walters, Jenny Wang, Lin Wang, Neil Wright, Ling Yang, Xiaoming Yang. **National Co-ordinating Centre, Beijing:** Yu Guo, Xiao Han, Can Hou, Jun Lv, Pei Pei, Chao Liu, Canqing Yu. **10 Regional Co-ordinating Centres: Qingdao CDC:** Zengchang Pang, Ruqin Gao, Shanpeng Li, Shaojie Wang, Yongmei Liu, Ranran Du, Yajing Zang, Liang Cheng, Xiaocao Tian, Hua Zhang, Yaoming Zhai, Feng Ning, Xiaohui Sun, Feifei Li. **Licang CDC:** Silu Lv, Junzheng Wang, Wei Hou. **Heilongjiang Provincial CDC:** Mingyuan Zeng, Ge Jiang, Xue Zhou. **Nangang CDC:** Liqiu Yang, Hui He, Bo Yu, Yanjie Li, Qinai Xu,Quan Kang, Ziyan Guo. **Hainan Provincial CDC:** Dan Wang, Ximin Hu, Jinyan Chen, Yan Fu, Zhenwang Fu, Xiaohuan Wang. **Meilan CDC:** Min Weng, Zhendong Guo, Shukuan Wu,Yilei Li, Huimei Li, Zhifang Fu. **Jiangsu Provincial CDC:** Ming Wu, Yonglin Zhou, Jinyi Zhou, Ran Tao, Jie Yang, Jian Su. **Suzhou CDC:** Fang liu, Jun Zhang, Yihe Hu, Yan Lu, , Liangcai Ma, Aiyu Tang, Shuo Zhang, Jianrong Jin, Jingchao Liu. **Guangxi Provincial CDC:** Zhenzhu Tang, Naying Chen, Ying Huang. **Liuzhou CDC:** Mingqiang Li, Jinhuai Meng, Rong Pan, Qilian Jiang, Jian Lan,Yun Liu, Liuping Wei, Liyuan Zhou, Ningyu Chen Ping Wang,

Fanwen Meng, Yulu Qin,, Sisi Wang. **Sichuan Provincial CDC:** Xianping Wu, Ningmei Zhang, Xiaofang Chen,Weiwei Zhou. **Pengzhou CDC:** Guojin Luo, Jianguo Li, Xiaofang Chen, Xunfu Zhong, Jiaqiu Liu, Qiang Sun. **Gansu Provincial CDC:** Pengfei Ge, Xiaolan Ren, Caixia Dong. **Maiji CDC:** Hui Zhang, Enke Mao, Xiaoping Wang, Tao Wang, Xi zhang. **Henan Provincial CDC:** Ding Zhang, Gang Zhou, Shixian Feng, Liang Chang, Lei Fan. **Huixian CDC:** Yulian Gao, Tianyou He, Huarong Sun, Pan He, Chen Hu, Xukui Zhang, Huifang Wu, Pan He. **Zhejiang Provincial CDC:** Min Yu, Ruying Hu, Hao Wang. Tongxiang CDC: Yijian Qian, Chunmei Wang, Kaixu Xie, Lingli Chen, Yidan Zhang, Dongxia Pan, Qijun Gu. **Hunan Provincial CDC:** Yuelong Huang, Biyun Chen, Li Yin, Huilin Liu, Zhongxi Fu, Qiaohua Xu. **Liuyang CDC:** Xin Xu, Hao Zhang, Huajun Long, Xianzhi Li, Libo Zhang, Zhe Qiu.

Supplementary Material 2. Questions about tea consumption and smoking in the CKB study.

Information on tea consumption was assessed by the following questions: (1) During the past 12 months, how often did you drink any tea (never or rarely, only occasionally, only at certain seasons, every month but less than weekly, usually at least once a week)? For those who answered "usually at least once a week", we further asked: (2) During the past 12 months, on how many days did you drink tea in a typical week (1-2 days/week, 3-5 days/week, daily or almost every day)? (3) At about what age did you start drinking tea almost weekly? (4) Which kind of tea do you drink mostly (green /jasmine tea, oolong tea, black tea, other tea), and how many cups do you usually drink on days when you drink tea (with 300 ml as a standard cup size)? (5) How often do you change tea leaves during a day? (6) How much tea leaves do you usually add each time (with a picture showing the amount of tea leaves in grams)? For those who answered other than "usually at least once a week" to question (1), we then asked: (7) In the past, did you ever have a period of at least 1 year during which you usually drank tea at least once a week?

Information on smoking was assessed by the following questions: (1) How often do you smoke tobacco now (do not smoke now, only occasionally, on most days, daily or almost every day)? (2) How much tobacco do you usually smoke? (filter cigarettes [number/day], non-filter cigarettes [number/day], hand-rolled cigarettes [liang/month], pipe or water pipe [liang/month], cigars [number/day]). For those who answered "on most days" or "daily or almost every day" to question (1), we then asked: (3) At about what age did you first start smoking on most days? For those who answered "do not smoke now" or "only occasionally" to question (1), we then asked: (4) In the past, how frequently did you smoke (did not smoke, smoked only occasionally, smoked on most days, smoked daily or almost every day) and how many years ago did you last stop smoking regularly?

Supplementary Table 1. Association between tea consumption and risk of PD according to sex.

	Men (n=210,202)			Women (n=302,521)			$p_{interaction}$
	Cases	Cases/PYs (/1000)	HRs (95%CIs)	Cases	Cases/PYs (/1000)	HRs (95%CIs)	
Tea consumption frequency and leaf grams							0.773
Never in life	104	0.25	1.00	222	0.15	1.00	
Less than daily	180	0.20	0.89 (0.69,1.15)	169	0.13	0.95 (0.76,1.19)	
Daily (all)	156	0.17	0.67 (0.50,0.89)	91	0.17	0.69 (0.50,0.95)	
Daily consumption (g/day)							
≤3.0	79	0.18	0.63 (0.45,0.88)	72	0.2	0.74 (0.53,1.05)	
>3.0	77	0.16	0.70 (0.51,0.97)	19	0.12	0.55 (0.33,0.91)	
Duration of consumption (y)							0.618
Never in life	104	0.25	1.00	222	0.15	1.00	
Daily consumption (y)							
<25	53	0.11	0.62 (0.42,0.90)	25	0.09	0.77 (0.48,1.22)	
≥25	103	0.24	0.68 (0.48,0.97)	66	0.26	0.58 (0.34,1.00)	
Types of tea consumed							0.252
Never in life	104	0.25	1.00	222	0.15	1.00	
Daily consumption							
Green tea	127	0.17	0.66 (0.47,0.92)	88	0.18	0.77 (0.49,1.18)	
Others	29	0.18	0.61 (0.37,1.01)	3	0.07	0.36 (0.11,1.15)	

CI, confidence interval; HR, hazard ratio; PD, Parkinson's disease; PYs, person years.

Multivariable analyses were adjusted for the same variables as model3 in Fig. 1 and were stratified jointly by regions and age at baseline in 5-year intervals.

Supplementary Table 2. Sensitivity analyses of the association between tea consumption and risk of PD.

	Never in life	Less than daily	Daily (all)	Daily consumption (grams/day)	
				≤3.0	>3.0
**Excluding cases identified during the first 2 years of follow-up (n=512,669)**					
Cases	298	330	240	146	94
Cases/PYs (/1000)	0.16	0.15	0.17	0.18	0.15
HRs (95%CIs)	1.00	0.93 (0.79,1.11)	0.71 (0.57,0.88)	0.71 (0.55,0.90)	0.72 (0.55,0.94)
**Excluding cases identified during the first 5 years of follow-up (n=512,494)**					
Cases	243	263	187	112	75
Cases/PYs (/1000)	0.13	0.12	0.13	0.14	0.12
HRs (95%CIs)	1.00	0.92 (0.76,1.12)	0.72 (0.56,0.91)	0.71 (0.54,0.93)	0.73 (0.54,0.98)
**Excluding participants with prevalent cancer, stroke, or coronary heart diseases at baseline (n=487,209)**					
Cases	276	308	221	137	84
Cases/PYs (/1000)	0.15	0.15	0.16	0.18	0.14
HRs (95%CIs)	1.00	0.92 (0.77,1.10)	0.68 (0.54,0.85)	0.68 (0.53,0.88)	0.67 (0.51,0.89)
**Excluding participants with a medical history of head injury at baseline (n=507,070)**					
Cases	324	341	246	151	95
Cases/PYs (/1000)	0.17	0.16	0.17	0.19	0.15
HRs (95%CIs)	1.00	0.91 (0.77,1.07)	0.69 (0.56,0.85)	0.69 (0.55,0.88)	0.68 (0.52,0.88)
**Further adjusting for intake of meat, fresh fruits, and vegetables (n=512,723)**					
Cases	326	349	247	151	96
Cases/PYs (/1000)	0.17	0.16	0.17	0.19	0.15
HRs (95%CIs)	1.00	0.92 (0.78,1.09)	0.69 (0.56,0.84)	0.69 (0.54,0.87)	0.68 (0.53,0.88)
**Further adjusting for the duration of storing pesticide at home (n=512,723)**					
Cases	326	349	247	151	96
Cases/PYs (/1000)	0.17	0.16	0.17	0.19	0.15
HRs (95%CIs)	1.00	0.92 (0.78,1.09)	0.68 (0.55,0.84)	0.68 (0.54,0.87)	0.68 (0.52,0.88)

CI, confidence interval; HR, hazard ratio; PD, Parkinson's disease; PYs, person years.

Multivariable analyses were adjusted for the same covariates as model3 in Fig. 1, as appropriate.

Supplementary Table 3. Subgroup analysis of associations between tea consumption and risk of PD according to potential baseline risk factors (n=512,723).

	Never in life		Less than daily		Daily		$p_{interaction}$
	Cases	HRs	Cases	HRs (95% CIs)	Cases	HRs (95% CIs)	
Age at baseline (y)							0.208
<50	29	1.00	42	0.86 (0.51,1.45)	33	0.90 (0.47,1.73)	
50-59	84	1.00	111	0.99 (0.72,1.36)	57	0.55 (0.36,0.83)	
≥60	213	1.00	196	0.91 (0.73,1.12)	157	0.72 (0.55,0.93)	
Region							0.851
Urban	173	1.00	173	0.92 (0.74,1.16)	85	0.64 (0.47,0.86)	
Rural	153	1.00	176	0.93 (0.72,1.20)	162	0.73 (0.54,0.99)	
Education							0.803
Middle school or higher	109	1.00	149	0.91 (0.70,1.18)	94	0.73 (0.53,1.00)	
Primary school or lower	217	1.00	200	0.93 (0.75,1.16)	153	0.66 (0.50,0.87)	
Alcohol consumption							0.442
Daily	14	1.00	41	1.08 (0.58,2.04)	38	0.65 (0.33,1.26)	
Less than daily	312	1.00	308	0.90 (0.76,1.08)	209	0.69 (0.56,0.87)	
Dairy products consumption							0.123
≥4 days/week	70	1.00	63	0.72 (0.50,1.04)	33	0.42 (0.26,0.66)	
<4 days/week	256	1.00	286	1.00 (0.83,1.21)	214	0.78 (0.62,0.99)	
Physical activity [a]							0.018
higher level	103	1.00	85	0.67 (0.49,0.92)	68	0.76 (0.51,1.12)	
lower level	223	1.00	264	1.04 (0.85,1.27)	179	0.67 (0.52,0.86)	
BMI (kg/m^2)							0.929
≥24.0	164	1.00	172	0.86 (0.68,1.09)	109	0.60 (0.44,0.80)	
<24.0	162	1.00	177	0.99 (0.78,1.25)	138	0.78 (0.58,1.06)	
Prevalent hypertension							0.855
Yes	164	1.00	156	0.87 (0.68,1.12)	125	0.64 (0.48,0.87)	
No	162	1.00	193	0.96 (0.76,1.21)	122	0.71 (0.53,0.95)	

	Never in life		Less than daily		Daily		$p_{interaction}$
	Cases	HRs	Cases	HRs (95% CIs)	Cases	HRs (95% CIs)	
Prevalent diabetes							0.421
Yes	37	1.00	27	0.73 (0.42,1.26)	21	0.62 (0.32,1.20)	
No	289	1.00	322	0.95 (0.79,1.13)	226	0.69 (0.56,0.87)	

BMI: body mass index; CI, confidence interval; HR, hazard ratio; PD, Parkinson's disease.

Multivariable analyses were adjusted for the same covariates as model3 in Fig. 1, as appropriate.

[a] METs were divided into two groups according to sex-specific median of total physical activity level.

Supplementary Table 4. Association between smoking and risk of PD according to sex.

	Men (n= 210,202)			Women (n= 302,521)			$p_{\text{interaction}}$
	Cases	Cases/PYs (/1000)	HRs (95%CIs)	Cases	Cases/PYs (/1000)	HRs (95%CIs)	
Smoking status							0.342
Never or occasional	161	0.28	1.00	464	0.14	1.00	
Former	104	0.36	0.99 (0.77,1.29)	5	0.19	0.57 (0.23,1.40)	
Current (all)	175	0.13	0.68 (0.54,0.86)	13	0.17	0.68 (0.38,1.21)	
Current smoker (cig or equivalent/day)							
<20	92	0.15	0.76 (0.58,0.99)	11	0.18	0.67 (0.36,1.25)	
≥20	83	0.11	0.60 (0.45,0.80)	2	0.16	0.71 (0.17,2.91)	
Duration of smoking							0.916
Never	161	0.28	1.00	464	0.14	1.00	
Current smoking (y)							
<30	52	0.07	0.83 (0.58,1.18)	4	0.11	0.65 (0.24,1.77)	
≥30	123	0.21	0.65 (0.50,0.84)	9	0.23	0.68 (0.34,1.36)	
							0.366
Years since stopped smoking							
Never or occasionally	161	0.28	1.00	464	0.14	100	
Stopped smoking (y)							
⩾6	79	0.46	1.17 (0.88,1.56)	4	0.24	0.67 (0.25,1.86)	
<6	25	0.22	0.67 (0.43,1.03)	1	0.10	0.34 (0.05,2.41)	

CI, confidence interval; HR, hazard ratio; PD, Parkinson's disease; PYs, person years.

Multivariable analyses were adjusted for the same variables as model3 in Figure2 and were stratified jointly by regions and age at baseline in 5-year intervals.

Supplementary Table 5. Sensitivity analyses of the association between smoking and risk of PD.

	Never or occasional	Former	Current (all)	Current smoking (cig or equivalent/day)	
				<20	≥20
**Excluding cases identified during the first 2 years of follow-up (n=512,669)**					
Cases	588	103	177	94	83
Cases/PYs (/1000)	0.16	0.33	0.12	0.14	0.11
HRs (95%CIs)	1.00	0.94 (0.74,1.21)	0.64 (0.52,0.80)	0.69 (0.53,0.88)	0.59 (0.45,0.78)
**Excluding cases identified during the first 5 years of follow-up (n=512,494)**					
Cases	478	81	134	73	61
Cases/PYs (/1000)	0.13	0.33	0.12	0.14	0.11
HRs (95%CIs)	1.00	0.88 (0.67,1.17)	0.59 (0.46,0.75)	0.64 (0.48,0.85)	0.53 (0.39,0.73)
**Excluding participants with prevalent cancer, stroke, or coronary heart diseases at baseline (n=487,209)**					
Cases	551	93	161	83	78
Cases/PYs (/1000)	0.15	0.33	0.12	0.13	0.10
HRs (95%CIs)	1.00	0.98 (0.75,1.27)	0.61 (0.48,0.77)	0.65 (0.49,0.85)	0.57 (0.42,0.76)
**Excluding participants with a medical history of head injury at baseline (n=507,070)**					
Cases	619	106	186	102	84
Cases/PYs (/1000)	0.16	0.34	0.13	0.15	0.11
HRs (95%CIs)	1.00	0.94 (0.74,1.20)	0.66 (0.53,0.82)	0.72 (0.57,0.93)	0.58 (0.44,0.76)
**Further adjusting for intake of meat, fresh fruits, and vegetables (n=512,723)**					
Cases	625	109	188	103	85
Cases/PYs (/1000)	0.16	0.35	0.13	0.15	0.11
HRs (95%CIs)	1.00	0.96 (0.75,1.22)	0.66 (0.53,0.82)	0.73 (0.57,0.93)	0.58 (0.44,0.77)
**Further adjusting for the duration of storing pesticide at home (n=512,723)**					
Cases	625	109	188	103	85
Cases/PYs (/1000)	0.16	0.35	0.13	0.15	0.11
HRs (95%CIs)	1.00	0.96 (0.75,1.22)	0.66 (0.53,0.82)	0.73 (0.57,0.93)	0.58 (0.44,0.77)

CI, confidence interval; HR, hazard ratio; PD, Parkinson's disease; PYs, person years.

Multivariable analyses were adjusted for the same covariates as model3 in Fig. 2, as appropriate.

Supplementary Table 6. Subgroup analysis of associations between smoking and risk of PD according to potential baseline risk factors (n=512,723).

	Never		Former		Current		$p_{interaction}$
	Cases	HRs	Cases	HRs (95% CIs)	Cases	HRs (95% CIs)	
Age at baseline (y)							0.848
<50	72	1.00	6	1.24 (0.48,3.22)	26	0.67 (0.35,1.29)	
50-59	183	1.00	17	0.72 (0.40,1.28)	52	0.58 (0.38,0.89)	
≥60	370	1.00	86	1.00 (0.76,1.32)	110	0.69 (0.53,0.90)	
Region							0.130
Urban	297	1.00	56	0.90 (0.65,1.25)	78	0.80 (0.59,1.09)	
Rural	328	1.00	53	1.03 (0.72,1.46)	110	0.57 (0.43,0.77)	
Education							0.289
Middle school or higher	219	1.00	48	0.96 (0.67,1.36)	85	0.82 (0.60,1.11)	
Primary school or lower	406	1.00	61	0.93 (0.67,1.29)	103	0.55 (0.41,0.74)	
Alcohol consumption							0.324
Daily	18	1.00	21	1.41 (0.71,2.81)	54	1.24 (0.67,2.28)	
Less than daily	607	1.00	88	0.94 (0.73,1.22)	134	0.59 (0.47,0.74)	
Dairy products consumption							0.140
≥4 days/week	107	1.00	34	1.50 (0.95,2.38)	25	0.91 (0.55,1.53)	
<4 days/week	518	1.00	75	0.82 (0.62,1.09)	163	0.61 (0.48,0.77)	
Physical activity [a]							0.210
higher level	174	1.00	28	1.14 (0.70,1.87)	54	0.57 (0.37,0.87)	
lower level	451	1.00	81	0.91 (0.69,1.19)	134	0.70 (0.55,0.90)	
BMI (kg/m^2)							0.216
≥24.0	309	1.00	58	0.93 (0.67,1.30)	78	0.74 (0.54,1.01)	
<24.0	316	1.00	51	0.98 (0.70,1.39)	110	0.62 (0.46,0.83)	
Prevalent hypertension							0.079
Yes	303	1.00	53	0.85 (0.60,1.20)	89	0.71 (0.52,0.96)	
No	322	1.00	56	1.10 (0.78,1.53)	99	0.63 (0.47,0.84)	

	Never		Former		Current		$p_{interaction}$
	Cases	HRs	Cases	HRs (95% CIs)	Cases	HRs (95% CIs)	
Prevalent diabetes							0.640
Yes	62	1.00	10	0.70 (0.32,1.51)	13	0.84 (0.41,1.73)	
No	563	1.00	99	1.00 (0.78,1.29)	175	0.66 (0.52,0.82)	

BMI: body mass index; CI, confidence interval; HR, hazard ratio; PD, Parkinson's disease.

Multivariable analyses were adjusted for the same covariates as model3 in Figure2, as appropriate.

[a] METs were divided into two groups according to sex-specific median of total physical activity level.
